# Burnout and life satisfaction among physicians working in intensive care units in Brazil: a nationwide workforce survey

**DOI:** 10.62675/2965-2774.20260445

**Published:** 2026-08-18

**Authors:** Ederlon Rezende, Cristiano Augusto Franke, Marcelo de Oliveira Maia, Mateus de Souza Ribeiro, Gabriel da Costa Medeiros de Souza, Alex Jones Flores Cassenote, Patricia Machado Veiga de Carvalho Mello

**Affiliations:** 1 Departamento de Medicina Intensiva Hospital do Servidor Público Estadual de São Paulo “Francisco Morato de Oliveira” São Paulo SP Brazil Departamento de Medicina Intensiva, Hospital do Servidor Público Estadual de São Paulo “Francisco Morato de Oliveira” - São Paulo (SP), Brazil.; 2 Unidade de Terapia Intensiva de Queimados e do Trauma Hospital de Pronto Socorro de Porto Alegre Porto Alegre RS Brazil Unidade de Terapia Intensiva de Queimados e do Trauma, Hospital de Pronto Socorro de Porto Alegre - Porto Alegre (RS), Brazil.; 3 Departamento de Terapia Intensiva Hospital Anchieta Taguatinga e Ceilândia Brasília DF Brazil Departamento de Terapia Intensiva, Hospital Anchieta Taguatinga e Ceilândia - Brasília (DF), Brazil.; 4 Faculdade Santa Marcelina São Paulo SP Brazil Faculdade Santa Marcelina - São Paulo (SP), Brazil.; 5 Faculdade de Medicina Universidade de São Paulo São Paulo SP Brazil Faculdade de Medicina, Universidade de São Paulo - São Paulo (SP), Brazil.; 6 Unidade de Terapia Intensiva Hospital de Terapia Intensiva Teresina PI Brazil Unidade de Terapia Intensiva, Hospital de Terapia Intensiva - Teresina (PI), Brazil.

**Keywords:** Critical care, Psychological well-being, Burnout, professional, Burnout, psychological, Personal satisfaction, Workforce, Occupational stress, Surveys and questionnaires, Intensive care units

## Abstract

**Objective:**

To estimate the prevalence of burnout and low life satisfaction and identify associated factors among physicians working in intensive care units in Brazil.

**Methods:**

We conducted a national, cross-sectional survey (March - October 2024) using probabilistic sampling stratified by specialist status. Eligible participants were physicians with ≥ 1 year of intensive care unit experience across Brazil’s 27 federative units. Burnout was measured with the Maslach Burnout Inventory-Human Services Survey (overall burnout defined as ≥ 1 altered domain), and life satisfaction with the Satisfaction With Life Scale (low life satisfaction < 25 points). Because many physicians hold multiple intensive care unit jobs, the unit of analysis was the physician-employment link. Prevalence ratios and 95% confidence intervals were estimated using multivariable Poisson regression with robust variance under a Generalized Estimating Equations framework.

**Results:**

Among 2,338 physicians (3,112 intensive care unit employment links), burnout prevalence was 68.9%, and low life satisfaction 54.2%. In adjusted analyses, burnout was less frequent among physicians aged > 50 years (prevalence ratio of 0.75; 95%CI 0.69 - 0.82) and those reporting frequent recovery behaviors, particularly good sleep quality ≥ 3 days/week (prevalence ratio of 0.81; 95%CI 0.77 - 0.86), while work schedule overload was positively associated with burnout (prevalence ratio of 1.25; 95%CI 1.15 - 1.36). Low life satisfaction was independently associated with working outside certified intensive care practice (prevalence ratio of 1.13; 95%CI 1.04 - 1.24), longer commuting distances (> 20km/week), and schedule overload, whereas leisure activity, adequate nutrition, and good sleep quality were inversely associated.

**Conclusion:**

Burnout and low life satisfaction are highly prevalent among intensive care unit physicians in Brazil. Organizational load, particularly schedule burden and commuting distance, and recovery behaviors show independent associations with one or both outcomes. These findings highlight opportunities for organization-directed workload reforms and for promoting access to recovery resources as strategies to support clinician well-being and the sustainability of the intensive care unit workforce.

## INTRODUCTION

Burnout and decreased well-being among physicians have become central concerns in critical care, where sustained cognitive load, high-acuity decision-making, and prolonged exposure to suffering contribute to psychological strain.^(1-3)^ International studies demonstrate consistently high burnout prevalence among intensivists, with implications for patient safety, workforce retention, and system resilience.^(4-8)^Although organizational factors such as staffing patterns, workload, night-shift burden, and commuting demands are key determinants of clinician well-being,^(9-13)^ data from low- and middle-income countries remain limited.

In Brazil, existing evidence shows important signs of psychological distress among intensive care unit (ICU) professionals, but current studies remain restricted in scope. A multicenter survey in five capitals reported high levels of burnout among intensivists but relied on convenience sampling.^(14)^ Another Brazilian study found high burnout prevalence during the coronavirus disease 2019 (COVID-19) pandemic among ICU physicians, but was limited to a specific context.^(15)^ These findings underscore the need for robust workforce-level data capable of capturing the heterogeneity of ICU practice across the country.

Brazil’s critical care workforce is characterized by diverse employment arrangements, multiple concurrent jobs, and marked regional disparities in ICU organization and resources.^(12)^ Despite these complexities, no nationally representative study has previously quantified burnout or life satisfaction among ICU physicians or examined how organizational load and recovery behaviors are associated with well-being at a population level.

This study aimed to estimate the prevalence of burnout and low life satisfaction and identify associated factors among physicians working in ICU in Brazil.

## METHODS

### Study design and setting

We conducted a national, cross-sectional survey between March and October 2024 to evaluate burnout and life satisfaction among ICU physicians in Brazil. The study used probabilistic sampling with national geographic coverage across the 27 federative units.

### Eligibility criteria

Eligible participants were physicians with ≥ 1 year of continuous ICU practice at the time of recruitment. Intensivists and non-intensivists working in adult, pediatric, neonatal, or specialized ICUs were included. Physicians without a valid e-mail address in the national medical registry were excluded because survey access required direct electronic authentication.

### Sampling strategy

A stratified probabilistic sampling design with replacement was used to enhance representativeness. Stratification was based on specialist registration (*Registro de Qualificação de Especialista* [RQE]) into three predefined groups:

–Physicians with RQE in intensive care medicine.
–Physicians with RQE in related areas (e.g., internal medicine, surgery, anesthesiology, cardiology, pulmonology).
–Physicians without RQE in these fields but working in ICUs.

Within each stratum, participants were randomly selected. Non-respondents were replaced after seven days. This strategy ensured proportionality across strata and reduced selection bias.

### Sample size

Sample size was calculated using a finite-population formula, considering the estimated national population of ICU physicians, a 95% confidence level, an expected proportion of 0.50 to maximize variance, and a margin of error compatible with national representativeness. The final sample included 2,338 physicians, contributing 3,112 physician-employment links.

### Data collection procedures

The *Conselho Federal de Medicina* distributed individualized invitations through a secure online platform. Before accessing the questionnaire, participants completed identity authentication using a unique validation code. Duplicate responses were prevented by linkage to this code. All data were stored in a secure environment administered by the council.

### Outcomes

#### Burnout

Burnout was assessed using the Maslach Burnout Inventory-Human Services Survey (MBI-HSS), examining the three core dimensions: emotional exhaustion, depersonalization, and personal accomplishment. Each domain was analyzed both as a continuous score and as a categorical variable using established cutoff values. Emotional exhaustion was classified as low (≤ 16), moderate (17 - 26), or high (≥ 27); depersonalization as low (≤ 6), moderate (7 - 9), or high (≥ 10); and personal accomplishment as high (≥ 39), moderate (34 - 38), or low (≤ 33). In addition to reporting the distribution of each dimension separately, we operationalized burnout using multiple aggregated definitions. Consistent with prior literature, overall burnout was defined as the presence of ≥ 1 altered domain (high emotional exhaustion, high depersonalization, or low personal accomplishment). We also classified burnout severity as partial burnout (one or two altered domains) and severe burnout (three altered domains simultaneously), allowing a graded interpretation of burnout burden and facilitating comparison across alternative definitions.

#### Life satisfaction

Life satisfaction was measured using the Satisfaction With Life Scale (SWLS).^(16)^ Scores < 25 were classified as low life satisfaction.

#### Covariates

Data collected encompassed sociodemographic and professional background characteristics (sex, age, year of graduation, type of medical school, region, and municipality of practice); education and professional qualifications (completion of intensive care medicine residency, specialist certification, RQE status, postgraduate training, and professional society memberships); labor market and employment conditions (types of employment contracts, including formal employment governed by Brazilian labor law, statutory employment, and independent contractor arrangements; weekly workload; shift characteristics; remuneration; and distribution of professional activities); and workplace characteristics (type of ICU, availability of resources, presence of multidisciplinary teams, clinical protocols, and patient flow processes). In addition, data on career perspectives were collected, including expectations regarding future workload, sectoral mobility, and intended retirement age.

## Statistical analysis

Descriptive statistics were presented as means (± standard deviation), medians (interquartile ranges), and proportions, as appropriate. Associations between outcomes and covariates were estimated using multivariable Poisson regression with robust variance under a Generalized Estimating Equations (GEE) framework to account for within-physician clustering across employment links. Prevalence ratios (PRs) and 95% confidence intervals (CIs) were reported. Analyses were performed using Stata version 19 (StataCorp, College Station, TX, USA).

The unit of analysis was the physician-employment link, reflecting each distinct ICU job held by an individual physician. Job-level exposures included workload characteristics, employment contract type, schedule organization, and commuting distance, all of which were measured separately for each ICU position. Physician-level characteristics, including age, sex, specialist status, and recovery behaviors, were measured once per individual and subsequently linked to all corresponding employment records. In analytical models, physician-level variables were therefore replicated across multiple job observations for the same physician. To account for the resulting within-physician correlation, we used GEE with robust variance estimators, specifying the physician as the clustering unit. This approach allowed simultaneous assessment of job-specific exposures and individual-level characteristics while preserving appropriate statistical inference.

Crude models were estimated for each covariate, and variables with p values < 0.20 in the bivariate analyses were considered candidates for multivariable modeling. Prior to and during multivariable model construction, collinearity among candidate covariates, particularly conceptually related workload variables, was assessed using variance inflation factors (VIF). Variables exhibiting substantial collinearity (VIF > 5) were not entered simultaneously in the same model; in such cases, the covariate with greater epidemiological interpretability and model stability was retained. Multivariable models were then constructed using a backward stepwise selection procedure guided by the Quasi-likelihood under the Independence Model Criterion (QIC), aiming to ensure model parsimony while maintaining appropriate control for confounding. Variables retained at a given stage remained in subsequent models regardless of statistical significance. Final models for each outcome included all covariates selected through this process, and prevalence ratios with 95% confidence intervals and associated p values were reported for each category, with the most epidemiologically relevant or most frequent category as the reference.

## Ethics

The study was reviewed and approved by the Research Ethics Committee of *Casa de Saúde Santa Marcelina*, under protocol number 40863020.0.0000.0066. Participation was voluntary, and informed electronic consent was obtained. No identifiable information was collected, and all procedures complied with national regulations and the Declaration of Helsinki.

## RESULTS

A total of 2,338 physicians working in ICUs across Brazil in 2024 were included. The mean age was 41.0 ± 11.0 years, with a mean time since graduation of 16.0 ± 12.0 years. Men comprised 60.7% of the sample and women 39.3%. Regarding career stage, 46.3% had graduated < 10 years prior, 23.8% between 10 - 20 years, and 29.9% > 20 years. Geographically, most respondents were registered in the Southeast (48.9%), followed by the Northeast (19.2%), the South (17.6%), the Central-West (9.1%), and the North (5.2%). Undergraduate training was evenly split between public (50.1%) and private (49.9%) institutions.

Regarding specialist status, 30.8% held RQE in Intensive Care Medicine (S1), 27.5% held RQE in related specialties (S2), and 41.8% had no RQE in Intensive Care Medicine or were certified in unrelated fields (S3). Partial dedication to intensive care was reported by 58.1% of participants, whereas 41.9% reported full-time dedication. Respondents reported 3,112 ICU employment contracts (mean 1.3 per physician): 73.2% held a single contract, 21.0% held two, 5.3% held three, and 0.5% held four (Table 1).

**Table 1:** Sociodemographic and professional characteristics of physicians working in intensive care units in Brazil, 2024

Variable	n (%)	95%CI
Sex		
Male	1,420 (60.7)	58.7 - 62.7
Female	918 (39.3)	37.3 - 41.3
Age (years; mean = 41.0 ± 11.0)		
< 35	843 (36.1)	34.1 - 38.0
35 - 50	978 (41.8)	39.8 - 43.8
> 50	517 (22.1)	20.5 - 23.8
Years since graduation (mean = 16.0 ± 12.0)		
< 10	1,083 (46.3)	44.3 - 48.4
10 - 20	556 (23.8)	22.1 - 25.5
> 20	698 (29.9)	28.0 - 31.7
Type of undergraduate medical school		
Public	1,143 (50.1)	48.0 - 52.1
Private	1,139 (49.9)	47.9 - 52.0
Region of registration		
North	122 (5.2)	4.4 - 6.2
Northeast	448 (19.2)	17.6 - 20.8
Southeast	1,144 (48.9)	46.9 - 51.0
South	411 (17.6)	16.1 - 19.2
Central-West	213 (9.1)	8.0 - 10.3
Professional strata (based on specialty status)		
S1: intensive care medicine	719 (30.8)	28.9 - 32.6
S2: related specialties	642 (27.5)	25.7 - 29.3
S3: No RQE / other specialties	977 (41.8)	39.8 - 43.8
Dedication to intensive care		
Partial	1,358 (58.1)	56.1 - 60.1
Full-time	980 (41.9)	39.9 - 43.9
Proportion of total working time dedicated to intensive care (%)		
Up to 10	238 (17.5)	15.6 - 19.6
10 - 30	417 (30.7)	28.3 - 33.2
30 - 50	371 (27.3)	25.0 - 29.7
50 - 70	249 (18.3)	16.3 - 20.5
70 - 90	83 (6.1)	4.9 - 7.5
Number of intensive care employment contracts		
1	1,712 (73.2)	71.4 - 75.0
2	490 (21.0)	19.3 - 22.6
3	124 (5.3)	4.5 - 6.3
4	12 (0.5)	0.3 - 0.9

Respondents were classified into three professional strata: S1 = formal certification in intensive care medicine; S2 = certification in related specialties (e.g., internal medicine, surgery, anesthesiology, cardiology, or pulmonology); and S3 = no certification in intensive care medicine (or related specialties) but currently working in intensive care units. Continuous variables are presented as mean ± standard deviation; categorical variables as n (%) with 95% confidence intervals. 95%CI - 95% confidence interval; RQE - certification in intensive care medicine.

### Burnout prevalence and dimensions

Burnout dimensions assessed by the MBI-HSS showed the following distributions: emotional exhaustion was low in 20.3%, moderate in 31.2%, and high in 48.5%; depersonalization was low in 54.4%, moderate in 29.6%, and high in 16.0%; personal accomplishment was low in 46.4%, moderate in 38.1%, and high in 15.5%. Overall burnout (≥ 1 altered domain) occurred in 68.9%, with 51.6% exhibiting partial burnout (1 - 2 altered domains) and 9.0% exhibiting severe burnout (all three domains altered).

Satisfaction With Life Scale score averaged 23.0 ± 7.0, with a median of 24.0 [18.0 - 28.0]. Categories were distributed as follows: extremely dissatisfied (5.0%), dissatisfied (9.7%), slightly dissatisfied (15.2%), neutral (24.3%), slightly satisfied (26.6%), satisfied (16.4%), and extremely satisfied (2.8%). Low life satisfaction (SWLS ≤ 24) occurred in 54.2% (Table 2).

**Table 2:** Burnout dimensions (categorical and continuous), overall, partial, and severe burnout, and life satisfaction among physicians working in intensive care units in Brazil, 2024 (n = 2,338)

Variable (mean ± SD; median [P25 - P75])	n (%)	95%CI
Emotional exhaustion (26.4 ± 10.6; 26.0 [18.0 - 34.0])		
Low	474 (20.3)	18.7 - 21.9
Moderate	729 (31.2)	29.3 - 33.1
High	1,135 (48.5)	46.5 - 50.6
Depersonalization (6.7 ± 5.4; 6.0 [2.0 - 10.0])		
Low	1,272 (54.4)	52.4 - 56.4
Moderate	692 (29.6)	27.8 - 31.5
High	374 (16.0)	14.6 - 17.5
Personal accomplishment (31.6 ± 6.8; 32.0 [28.0 - 37.0])		
Low	1,085 (46.4)	44.4 - 48.4
Moderate	890 (38.1)	36.1 - 40.0
High	363 (15.5)	14.1 - 17.0
Overall burnout (≥ 1 altered domain)	1,610 (68.9)	67.0 - 70.7
Partial burnout (1 - 2 altered domains)	1,206 (51.6)	49.6 - 53.6
Severe burnout (3 altered domains)	210 (9.0)	7.9 - 10.2
Satisfaction With Life Scale (23.0 ± 7.0; 24.0 [18.0 - 28.0])		
Extremely dissatisfied	116 (5.0)	4.1 - 5.9
Dissatisfied	227 (9.7)	8.6 - 11.0
Slightly dissatisfied	356 (15.2)	13.8 - 16.7
Neutral	568 (24.3)	22.6 - 26.1
Slightly satisfied	621 (26.6)	24.8 - 28.4
Satisfied	384 (16.4)	15.0 - 18.0
Extremely satisfied	66 (2.8)	2.2 - 3.6
Low satisfaction		
Yes	1,267 (54.2)	52.2 - 56.2
No	1,071 (45.8)	43.8 - 47.8

Emotional exhaustion, depersonalization, and personal accomplishment were assessed using the Maslach Burnout Inventory – Human Services Survey (MBI-HSS). “Overall burnout” was defined as the presence of at least one altered domain (high emotional exhaustion, high depersonalization, or low personal accomplishment). “Partial burnout” was defined as the presence of one or two altered domains, and “severe burnout” as the simultaneous alteration of all three domains. Life satisfaction was assessed using the Satisfaction With Life Scale, with total scores ranging from 5 to 35; interpretive categories are as follows: extremely dissatisfied (5 - 9), dissatisfied (10 - 14), slightly dissatisfied (15 - 19), neutral (20 - 24), slightly satisfied (25 - 29), satisfied (30 - 34), and extremely satisfied (35). Scores < 25 were classified as low satisfaction. All percentages were calculated based on complete cases. Values are expressed as counts n (%) with 95% confidence intervals for categorical variables, and as mean ± standard deviation and median [25^th^ - 75th percentiles] for continuous variables. 95%CI - 95% confidence interval.

### Associations with burnout

Burnout was more frequent among S2 and S3 groups than among certified intensivists (S1), more frequent in women than in men, and less common among physicians with > 20 years since graduation than among those with < 10 years. Burnout prevalence was also higher among those reporting work overload and lower among those engaging ≥ 3 days/week in physical activity, social activity, and leisure activities, maintaining adequate nutrition, and reporting good sleep quality.

In the adjusted model (Table 3), burnout was less frequent among physicians aged >50 years than among those aged < 35 years (PR = 0.749; 95%CI 0.686 - 0.818). Work schedule burden showed strong associations: “adequate, can expand” (PR = 0.903; 95%CI 0.834 - 0.978) and “overload” (PR = 1.251; 95%CI 1.151 - 1.360) compared with “reduced.” Higher income was associated with lower burnout (R$24,001 - 30,000: PR = 0.861; 95%CI 0.773 - 0.958; ≥ R$30,000: PR = 0.829; 95%CI 0.741 - 0.926, *versus* ≤ R$12,000). Recovery behaviors remained protective: ≥ 3 days/week of physical activity (PR = 0.907; 95%CI 0.861 - 0.957), ≥ 3 days/week of social activity (PR = 0.917; 95%CI 0.863 - 0.974), and ≥ 3 days/week of good sleep quality (PR = 0.814; 95%CI 0.773 - 0.856).

**Table 2:** Prevalence of burnout, crude and adjusted prevalence ratios according to sociodemographic, professional, and behavioral characteristics of physicians working in intensive care units in Brazil, 2024 (n = 2,338)

	**Prevalence**		**Crude**			**Adjusted**		
	**n (%)**	**95%CI**	**PR**	**95%CI**	**p value**	**PR**	**95%CI**	**p value**
Professional strata								
S1: intensive care medicine (ref.)	687 (63.0)	60.1 - 65.9	-	-	-	-	-	-
S2: related specialties	535 (68.4)	65.0 - 71.6	1.092	1.014 - 1.176	0.020	-	-	-
S3: No RQE/other specialties	900 (72.6)	70.0 - 75.0	1.177	1.103 - 1.255	< 0.001	-	-	-
Sex								
Female	857 (70.8)	68.2 - 73.4	1.085	1.029 - 1.144	0.003	-	-	-
Male (ref.)	1,265 (66.5)	64.3 - 68.6	-	-	-	-	-	-
Years since graduation								
< 10 (ref.)	978 (74.5)	72.0 - 76.8	-	-	-	-	-	-
10 - 20	558 (70.5)	67.2 - 73.7	0.952	0.896 - 1.011	0.108	-	-	-
> 20	539 (57.5)	54.3 - 60.7	0.749	0.697 - 0.805	< 0.001	-	-	-
Age group (years)								
< 35 (ref.)	806 (74.0)	71.3 - 76.6	-	-	-	-	-	-
35 - 50	949 (71.1)	68.6 - 73.5	0.963	0.912 - 1.016	0.167	1.001	0.947 - 1.057	0.979
> 50	367 (53.3)	49.5 - 57.1	0.708	0.648 - 0.773	< 0.001	0.749	0.686 - 0.818	< 0.001
Registration region								
North (ref.)	98 (64.1)	55.9 - 71.5	-	-	-	-	-	-
Northeast	410 (64.9)	61.0 - 68.6	0.968	0.845 - 1.109	0.641	-	-	-
Southeast	1,024 (68.3)	65.8 - 70.6	0.998	0.880 - 1.132	0.974	-	-	-
South	381 (70.0)	66.0 - 73.8	1.033	0.903 - 1.181	0.640	-	-	-
Central-West	209 (73.9)	68.3 - 78.8	1.093	0.948 - 1.261	0.220	-	-	-
Intensive care medicine residency								
Currently attending (ref.)	204 (86.1)	80.9 - 90.1	-	-	-	-	-	-
Already attended	501 (67.5)	64.0 - 70.9	0.773	0.714 - 0.837	< 0.001	-	-	-
Not attending/never attended	1,417 (66.4)	64.4 - 68.4	0.761	0.713 - 0.811	< 0.001	-	-	-
Dedication level								
Partial (ref.)	1,130 (69.9)	67.6 - 72.1	-	-	-	-	-	-
Full-time	992 (66.4)	63.9 - 68.7	0.961	0.911 - 1.015	0.154	0.948	0.889 - 1.011	0.103
Active ICU jobs								
1 (ref.)	1,191 (69.6)	67.3 - 71.7	-	-	-	-	-	-
2	666 (68.0)	64.9 - 70.9	0.959	0.898 - 1.024	0.208	-	-	-
3	237 (63.7)	58.6 - 68.6	0.940	0.838 - 1.053	0.283	-	-	-
4+	28 (58.3)	43.3 - 72.1	0.902	0.618 - 1.317	0.595	-	-	-
Travels outside the municipality								
No (ref.)	1,478 (68.2)	66.2 - 70.1	-	-	-	-	-	-
Yes	644 (68.2)	65.1 - 71.2	1.011	0.954 - 1.072	0.705	-	-	-
Weekly distance traveled (km)								
≤ 20 (ref.)	850 (68.4)	65.8 - 71.0	-	-	-	-	-	-
21 - 100	835 (70.0)	67.3 - 72.6	1.040	0.981 - 1.103	0.187	-	-	-
> 100	436 (64.5)	60.7 - 68.1	0.976	0.904 - 1.053	0.527	-	-	-
Work schedule type								
Reduced (ref.)	378 (70.5)	66.4 - 74.3	-	-	-	-	-	-
Adequate, can expand	601 (60.0)	56.9 - 63.1	0.866	0.801 - 0.937	< 0.001	0.903	0.834 - 0.978	0.012
Adequate, cannot expand	627 (65.0)	61.9 - 68.0	0.931	0.862 - 1.005	0.068	0.996	0.918 - 1.080	0.915
Overload	516 (84.5)	81.3 - 87.2	1.206	1.126 - 1.291	< 0.001	1.251	1.151 - 1.360	< 0.001
ICU type								
Adult (ref.)	1,792 (68.2)	66.4 - 70.0	-	-	-	-	-	-
Pediatric	154 (68.1)	61.6 - 74.1	0.989	0.823 - 1.189	0.906	-	-	-
Neonatal	49 (67.1)	55.0 - 77.4	0.984	0.798 - 1.213	0.878	-	-	-
Specialized	127 (67.9)	60.6 - 74.4	1.008	0.968 - 1.049	0.713	-	-	-
Medical director								
No (ref.)	1,861 (70.2)	68.4 - 71.9	-	-	-	-	-	-
Yes	261 (56.6)	51.9 - 61.2	1.115	1.059 - 1.174	< 0.001	-	-	-
Overnight shifts (days)								
No shifts (ref.)	338 (60.1)	55.9 - 64.2	-	-	-	-	-	-
< 3	1,219 (69.7)	67.5 - 71.8	1.163	1.075 - 1.259	< 0.001	-	-	-
≥ 3	565 (70.5)	67.2 - 73.7	1.161	1.073 - 1.257	< 0.001	-	-	-
Contract type								
Resident scholarship (ref.)	80 (87.0)	77.9 - 92.8	-	-	-	-	-	-
Employee	322 (69.0)	64.5 - 73.1	1.071	1.019 - 1.125	0.007	-	-	-
Contractor	1,131 (68.3)	66.0 - 70.6	1.075	1.025 - 1.128	0.003	-	-	-
Cooperative	198 (66.4)	60.7 - 71.7	1.081	1.025 - 1.141	0.004	-	-	-
Statutory	182 (63.6)	57.7 - 69.2	1.094	1.036 - 1.155	0.001	-	-	-
Other	197 (67.0)	61.3 - 72.3	1.068	1.011 - 1.129	0.019	-	-	-
Service type								
Private (ref.)	1,028 (66.6)	64.2 - 69.0	-	-	-	-	-	-
Public	1,094 (69.7)	67.4 - 72.0	0.988	0.978 - 0.998	0.018	-	-	-
Weekly workload (hours)								
≤ 20 (ref.)	427 (68.6)	64.8 - 72.2	-	-	-	-	-	-
20 - 40	727 (68.5)	65.6 - 71.3	0.992	0.924 - 1.063	0.812	-	-	-
40 - 60	516 (62.9)	59.4 - 66.1	0.916	0.844 - 0.994	0.036	-	-	-
60 - 80	317 (71.7)	67.2 - 75.8	1.065	0.978 - 1.160	0.148	-	-	-
≥ 80	135 (81.3)	74.4 - 86.8	1.163	1.044 - 1.296	0.006	-	-	-
Total monthly income (reais)								
≤ R$12,000 (ref.)	687 (73.2)	70.3 - 76.0	-	-	-	-	-	-
R$12,001 - 16,000	325 (68.0)	63.6 - 72.1	0.908	0.835 - 0.987	0.023	0.940	0.867 - 1.019	0.135
R$16,001 - 20,000	269 (72.9)	68.0 - 77.3	0.973	0.893 - 1.061	0.539	0.997	0.910 - 1.093	0.956
R$20,001 - 24,000	195 (66.1)	60.4 - 71.4	0.944	0.855 - 1.042	0.254	0.923	0.833 - 1.023	0.129
R$24,001 - 30,000	218 (62.1)	56.8 - 67.2	0.848	0.764 - 0.941	0.002	0.861	0.773 - 0.958	0.006
≥ R$30,000	265 (59.2)	54.4 - 63.7	0.838	0.758 - 0.925	< 0.001	0.829	0.741 - 0.926	< 0.001
Prefer not to answer	163 (70.0)	63.6 - 75.7	0.979	0.890 - 1.077	0.661	0.997	0.910 - 1.093	0.948
Physical activity (days)								
< 3 (ref.)	1,070 (74.5)	72.1 - 76.7	-	-	-	-	-	-
≥ 3	1,052 (62.8)	60.4 - 65.1	0.841	0.797 - 0.886	< 0.001	0.907	0.861 - 0.957	< 0.001
Leisure activity (days)								
< 3 (ref.)	1,249 (74.0)	71.8 - 76.1	-	-	-	-	-	-
≥ 3	873 (61.3)	58.7 - 63.8	0.834	0.789 - 0.881	< 0.001	-	-	-
Social activity (days)								
< 3 (ref.)	1,438 (73.5)	71.5 - 75.4	-	-	-	-	-	-
≥ 3	684 (59.2)	56.3 - 62.1	0.810	0.762 - 0.860	< 0.001	0.917	0.863 - 0.974	0.005
Adequate nutrition (days)								
< 3 (ref.)	631 (83.1)	80.2 - 85.7	-	-	-	-	-	-
≥ 3	1,491 (63.4)	61.4 - 65.3	0.762	0.725 - 0.800	< 0.001	-	-	-
Good sleep quality (days)								
< 3 (ref.)	888 (83.4)	81.0 - 85.5	-	-	-	-	-	-
≥ 3	1,234 (60.3)	58.1 - 62.4	0.730	0.695 - 0.767	< 0.001	0.814	0.773 - 0.856	< 0.001

The unit of analysis in all prevalence estimates and regression models was the physician-employment relationship, meaning that physicians with multiple active intensive care unit contracts contributed one observation for each employment link. Prevalence ratios and their respective 95%CI were estimated using Poisson regression with robust variance under a Generalized Estimating Equations approach to account for correlation between observations from the same individual. Crude prevalence ratios correspond to univariate models, while adjusted prevalence ratios were derived from a multivariable model including all variables with p < 0.20 in the bivariate analyses. Final model selection was performed using a backward stepwise procedure guided by the Quasi-likelihood under the Independence Model Criterion. The outcomes analyzed in the tables were burnout and low life satisfaction. Categories marked as (ref.) indicate the reference category in the regression models. Dashes (-) denote variables or categories that were not retained in the final adjusted model according to the stepwise selection criteria (e.g., lack of contribution to model fit or collinearity), or instances where adjusted estimates were not applicable. Percentages may not total 100% due to rounding. Variables related to physical activity, leisure activity, social activity, adequate nutrition, and good sleep quality refer to the number of days per week each behavior was reported (< 3 *versus* ≥ 3). Variables not retained in final models were excluded due to lack of independent association after multivariable adjustment or collinearity with conceptually related covariates. 95%CI - 95% confidence intervals; PR - prevalence ratios; RQE - certification in intensive care medicine; ICU - intensive care unit.

### Associations with low life satisfaction

Low life satisfaction followed similar patterns, being more frequent among S2 and S3 groups than S1, among younger physicians, and among those with heavier work schedules, longer weekly commuting distances, and greater total weekly workload. Frequent leisure activities, social activities, adequate nutrition, and good sleep quality were associated with lower prevalence.

In adjusted analyses (Table 4), low life satisfaction remained higher among physicians without RQE in intensive care medicine or a related specialty (S3 *versus* S1: PR = 1.133; 95%CI 1.038 - 1.237), among those with weekly travel distances of 21 - 100 km (PR = 1.150; 95%CI 1.073 - 1.232) and > 100 km (PR = 1.103; 95%CI 1.013 - 1.202) *versus* ≤ 20 km, and among those reporting schedule overload (PR = 1.241; 95%CI 1.130 - 1.363) versus a reduced schedule. Inverse associations were observed for leisure activity ≥ 3 days/week (PR = 0.857; 95%CI 0.800 - 0.918), adequate nutrition ≥ 3 days/week (PR = 0.879; 95%CI 0.820 - 0.942), and good sleep quality ≥ 3 days/week (PR = 0.749; 95%CI 0.699 - 0.803).

**Table 4:** Prevalence of low life satisfaction and crude and adjusted prevalence ratios according to sociodemographic, professional, and behavioral characteristics of physicians working in intensive care units in Brazil, 2024 (n = 2,338)

	Prevalence		Crude			Adjusted		
	n	95%CI	PR	95%CI	p value	PR	95%CI	p value
Professional strata								
S1: intensive care medicine (ref.)	1,090 (51.9)	49.0 - 54.9	-	-	-	-	-	-
S2: Related specialties	782 (58.2)	54.7 - 61.6	1.093	0.995 - 1.200	0.063	1.021	0.926 - 1.127	0.670
S3: No RQE/other specialties	1,240 (69.8)	67.1 - 72.3	1.123	1.020 - 1.235	0.018	1.133	1.038 - 1.237	0.005
Sex								
Female	1,210 (63.6)	60.8 - 66.2	1.078	1.011 - 1.148	0.022	-	-	-
Male (ref.)	1,902 (58.7)	56.5 - 60.9	-	-	-	-	-	-
Years since graduation (years)								
< 10 (ref.)	1,313 (67.8)	65.2 - 70.3	-	-	-	-	-	-
10 - 20	791 (57.9)	54.4 - 61.3	0.848	0.783 - 0.918	< 0.001	-	-	-
> 20	937 (52.7)	49.5 - 55.9	0.769	0.710 - 0.833	< 0.001	-	-	-
Age group (years)								
< 35 (ref.)	1,089 (66.9)	64.1 - 69.7	-	-	-	-	-	-
35 - 50	1,335 (61.1)	58.5 - 63.7	0.919	0.860 - 0.982	0.012	1.033	0.965 - 1.106	0.346
> 50	688 (49.6)	45.8 - 53.3	0.720	0.653 - 0.794	< 0.001	0.863	0.780 - 0.955	0.004
Registration region								
North (ref.)	153 (61.4)	53.5 - 68.8	-	-	-	-	-	-
Northeast	632 (59.5)	55.6 - 63.3	0.937	0.804 - 1.090	0.404	-	-	-
Southeast	1,500 (61.9)	59.4 - 64.3	0.975	0.846 - 1.123	0.721	-	-	-
South	544 (54.2)	50.0 - 58.4	0.865	0.739 - 1.012	0.073	-	-	-
Central-West	283 (68.2)	62.6 - 73.3	1.079	0.918 - 1.268	0.357	-	-	-
Intensive Care Medicine residency								
Currently attending (ref.)	237 (79.7)	74.2 - 84.4	-	-	-	-	-	-
Already attended	742 (52.7)	49.1 - 56.3	0.659	0.591 - 0.735	< 0.001	-	-	-
Not attending/never attended	2,133 (61.2)	59.1 - 63.3	0.767	0.706 - 0.834	< 0.001	-	-	-
Dedication level								
Partial (ref.)	1,617 (60.7)	58.3 - 63.1	-	-	-	-	-	-
Full-time	1,495 (60.5)	58.0 - 62.9	1.002	0.940 - 1.067	0.947	-	-	-
Active ICU jobs								
1 (ref.)	1,712 (61.3)	58.9 - 63.6	-	-	-	-	-	-
2	980 (60.8)	57.7 - 63.8	0.981	0.909 - 1.060	0.612	-	-	-
3	372 (57.3)	52.2 - 62.2	0.902	0.784 - 1.039	0.149	-	-	-
4+	48 (58.3)	44.3 - 71.2	0.918	0.595 - 1.420	0.699	-	-	-
Travels outside the municipality								
No (ref.)	2,168 (58.4)	56.3 - 60.5	-	-	-	-	-	-
Yes	944 (65.7)	62.6 - 68.6	1.099	1.028 - 1.177	0.006	-	-	-
Weekly distance traveled (km)								
≤ 20 (ref.)	1,242 (56.1)	53.3 - 58.9	-	-	-	-	-	-
21 - 100	1,193 (63.8)	61.0 - 66.5	1.151	1.072 - 1.232	< 0.001	1.150	1.073 - 1.232	< 0.001
> 100	676 (63.2)	59.5 - 66.7	1.100	1.007 - 1.200	0.034	1.103	1.013 - 1.202	0.024
Work schedule type								
Reduced (ref.)	536 (60.6)	56.4 - 64.7	-	-	-	-	-	-
Adequate, can expand	1,001 (54.9)	51.8 - 58.0	0.908	0.827 - 0.997	0.042	0.914	0.833 - 1.003	0.058
Adequate, cannot expand	964 (57.0)	53.8 - 60.0	0.915	0.833 - 1.010	0.066	0.974	0.885 - 1.073	0.597
Overload	611 (75.6)	72.1 - 78.9	1.263	1.159 - 1.376	< 0.001	1.241	1.130 - 1.363	< 0.001
ICU type								
Adult (ref.)	2,626 (60.7)	58.8 - 62.5	-	-	-	-	-	-
Pediatric	187 (60.4)	53.3 - 67.2	1.000	0.998 - 1.000	0.808	-	-	-
Neonatal	73 (57.5)	46.1 - 68.2	1.000	0.984 - 1.020	0.959	-	-	-
Specialized	226 (61.1)	54.6 - 67.2	1.000	0.987 - 1.010	0.946	-	-	-
Medical director								
No (ref.)	2,651 (63.6)	61.7 - 65.4	-	-	-	-	-	-
Yes	461 (43.6)	39.1 - 48.2	0.991	0.989 - 0.994	< 0.001	0.992	0.987 - 0.997	0.001
Overnight shifts (days)								
No shifts (ref.)	562 (48.0)	43.9 - 52.2	-	-	-	-	-	-
< 3	1,749 (61.1)	58.8 - 63.3	1.276	1.156 - 1.409	< 0.001	1.090	0.994 - 1.197	0.068
≥ 3	801 (68.4)	65.1 - 71.5	1.287	1.167 - 1.420	< 0.001	1.093	0.996 - 1.199	0.061
Contract type								
Resident scholarship (ref.)	92 (88.0)	79.8 - 93.2	-	-	-	-	-	-
Employee	467 (60.6)	56.1 - 64.9	0.997	0.995 - 0.998	< 0.001	-	-	-
Contractor	1,655 (59.6)	57.3 - 62.0	0.996	0.995 - 0.998	< 0.001	-	-	-
Cooperative	298 (61.4)	55.8 - 66.8	0.997	0.995 - 0.999	0.001	-	-	-
Statutory	286 (54.5)	48.8 - 60.2	0.996	0.994 - 0.998	< 0.001	-	-	-
Other	294 (62.9)	57.3 - 68.2	0.998	0.996 - 1.000	0.021	-	-	-
Service type								
Private (ref.)	1,543 (58.8)	56.3 - 61.2	-	-	-	-	-	-
Public	1,569 (62.4)	60.0 - 64.8	1.001	1.000 - 1.002	0.033	-	-	-
Weekly workload (hours)								
≤ 20 (ref.)	622 (56.4)	52.5 - 60.3	-	-	-	-	-	-
20 - 40	1,061 (59.2)	56.2 - 62.1	1.019	0.933 - 1.113	0.676	-	-	-
40 - 60	821 (58.8)	55.4 - 62.1	1.043	0.948 - 1.147	0.391	-	-	-
60 - 80	442 (65.6)	61.1 - 69.9	1.156	1.041 - 1.284	0.007	-	-	-
≥ 80	166 (80.7)	74.1 - 86.0	1.348	1.193 - 1.524	< 0.001	-	-	-
Total monthly income (reais)								
≤ R$12,000 (ref.)	938 (64.1)	61.0 - 67.1	-	-	-	-	-	-
R$12,001 - 16,000	478 (64.0)	59.6 - 68.2	0.978	0.890 - 1.074	0.635	0.989	0.901 - 1.086	0.817
R$16,001 - 20,000	369 (70.7)	65.9 - 75.1	1.064	0.967 - 1.173	0.203	1.060	0.959 - 1.173	0.254
R$20,001 - 24,000	295 (60.7)	55.0 - 66.1	0.959	0.852 - 1.081	0.485	0.947	0.841 - 1.067	0.370
R$24,001 - 30,000	351 (53.8)	48.6 - 59.0	0.865	0.767 - 0.976	0.019	0.864	0.762 - 0.981	0.024
≥ R$30,000	448 (46.9)	42.3 - 51.5	0.709	0.620 - 0.811	< 0.001	0.701	0.609 - 0.806	< 0.001
Prefer not to answer	233 (60.1)	53.7 - 66.2	0.967	0.859 - 1.090	0.578	0.957	0.858 - 1.069	0.437
Physical activity (days)								
< 3 (ref.)	1,437 (67.3)	64.8 - 69.7	-	-	-	-	-	-
≥ 3	1,675 (54.9)	52.5 - 57.2	0.806	0.756 - 0.858	< 0.001	-	-	-
Leisure activity (days)								
< 3 (ref.)	1,688 (69.8)	67.6 - 72.0	-	-	-	-	-	-
≥ 3	1,424 (49.6)	47.1 - 52.2	0.721	0.673 - 0.771	< 0.001	0.857	0.800 - 0.918	< 0.001
Social activity (days)								
< 3 (ref.)	1,957 (67.3)	65.2 - 69.3	-	-	-	-	-	-
≥ 3	1,155 (49.3)	46.4 - 52.1	0.738	0.686 - 0.794	< 0.001	-	-	-
Adequate nutrition (days)								
< 3 (ref.)	759 (80.1)	77.1 - 82.8	-	-	-	-	-	-
≥ 3	2,353 (54.3)	52.3 - 56.3	0.654	0.618 - 0.692	< 0.001	0.879	0.820 - 0.942	< 0.001
Good sleep quality (days)								
< 3 (ref.)	1,065 (81.1)	78.7 - 83.4	-	-	-	-	-	-
≥ 3	2,047 (49.9)	47.8 - 52.1	0.617	0.582 - 0.654	< 0.001	0.749	0.699 - 0.803	< 0.001

The unit of analysis in all prevalence estimates and regression models was the physician-employment relationship, meaning that physicians with multiple active intensive care unit contracts contributed one observation for each employment link. Prevalence ratios and their respective 95%CI were estimated using Poisson regression with robust variance under a Generalized Estimating Equations approach to account for correlation between observations from the same individual. Crude prevalence ratios correspond to univariate models, while adjusted prevalence ratios were derived from a multivariable model including all variables with p < 0.20 in the bivariate analyses. Final model selection was performed using a backward stepwise procedure guided by the Quasi-likelihood under the Independence Model Criterion. The outcomes analyzed in the tables were burnout and low life satisfaction. Categories marked as (ref.) indicate the reference category in the regression models. Dashes (-) denote variables or categories that were not retained in the final adjusted model according to the stepwise selection criteria (e.g., lack of contribution to model fit or collinearity), or instances where adjusted estimates were not applicable. Percentages may not total 100% due to rounding. Variables related to physical activity, leisure activity, social activity, adequate nutrition, and good sleep quality refer to the number of days per week each behavior was reported (< 3 *versus* ≥ 3). Variables not retained in final models were excluded due to lack of independent association after multivariable adjustment or collinearity with conceptually related covariates. 95%CI - 95% confidence intervals; PR - prevalence ratios; RQE - certification in intensive care medicine; ICU - intensive care unit.

## DISCUSSION

This nationwide probability survey demonstrates a substantial burden of burnout and low life satisfaction among ICU physicians in Brazil. Nearly seven out of ten physicians had at least one altered MBI-HSS domain, and more than half reported low life satisfaction. These estimates provide the most representative assessment of ICU physicians’ well-being nationwide.

The prevalence observed aligns with international evidence showing high psychological distress among intensivists, particularly where workloads, multiple contracts, and limited recovery time are common.^(4-8)^Large observational and interventional studies have consistently reported associations between work organization, including staffing patterns, workload intensity, and perceived schedule manageability, and emotional exhaustion and decreased well-being.^(9-13)^ Brazilian data, although previously limited, also documented significant burnout among intensivists in multicenter convenience studies^(14)^ and during pandemic-related strain.^(15)^ The present study expands this evidence by generating nationally representative estimates with a stratified probabilistic design. Accordingly, the prevalence reported here should be interpreted as reflecting the burden of burnout-related symptomatology rather than a diagnostic estimate of the syndrome.

The adjusted associations underscore the relevance of work organization. Physicians reporting schedule overload exhibited worse outcomes, whereas adequate, flexible schedules were inversely associated with burnout—aligning with international evidence on the detrimental effects of excessive workload.^(8,9,12)^Commuting distance also showed a graded association with life satisfaction, consistent with the literature, which demonstrates that long commuting times reduce subjective well-being and contribute to cumulative stress.^(17-19)^

Recovery behaviors were consistently associated with more favorable outcomes, although the behaviors retained in the adjusted models differed by outcome. Frequent physical activity, social activity, and good sleep quality were inversely associated with burnout, whereas frequent leisure activity, adequate nutrition, and good sleep quality were inversely associated with low life satisfaction. Similar associations have been reported in studies evaluating physical activity, social support, and sleep quality among physicians and healthcare professionals.^(20-23)^ These findings reflect a growing body of evidence showing that restorative behaviors may help mitigate the effects of organizational stressors.^(24-27)^

Specialist status was also associated with the outcomes. Physicians without RQE in intensive care medicine or a related specialty had a higher prevalence of low life satisfaction in the adjusted model, while higher burnout among S2 and S3 was observed only in crude analyses. These differences may reflect variation in training environments, decision-support structures, and workload distribution.

The lower prevalence of burnout among older physicians (> 50 years) mirrors findings from international physician surveys and may reflect greater role stability, experience, and improved coping over the course of a career.^(10,11)^

These findings have important implications for workforce planning and health policy. The observed associations suggest that clinician distress is linked to organizational and system-level characteristics, including workload intensity, fragmented employment, and commuting burden.^(8,12-22)^Although these factors are often discussed as potentially amenable to organizational or system-level interventions, causal inferences cannot be drawn from the present cross-sectional design.

Strategies such as standardizing staffing models, improving schedule predictability, and limiting excessive job accumulation have been proposed in the literature as potential approaches to addressing physician distress.^(24,26,27)^ Institutional investment in recovery-promoting environments, including nutrition, rest infrastructure, sleep hygiene, and opportunities for physical and social activities, is supported by evidence from controlled and quasi-experimental studies. However, their effectiveness may vary according to context.^(28,29)^Given the central role of intensivists in high-complexity care, physician well-being represents a critical dimension of workforce sustainability. Burnout is now recognized by the World Health Organization as an occupational phenomenon associated with chronic workplace stress that has not been successfully managed, reinforcing the relevance of organizational approaches to physician well-being.^(30)^ While the present study does not allow causal inference, it provides robust, nationally representative evidence describing how burnout and life satisfaction are patterned across organizational and individual characteristics among ICU physicians in Brazil. These findings should therefore be interpreted as descriptive and hypothesis-generating, supporting future longitudinal research and informing policy discussions aimed at sustaining intensive care medicine in the country.

Strengths of this study include its national scope; probabilistic sampling stratified by specialist status; the use of validated instruments to assess burnout (MBI-HSS) and life satisfaction (SWLS); modeling at the physician-employment link level with appropriate accounting for within-physician correlation; and the parallel evaluation of organizational and behavioral exposures. Limitations include the cross-sectional design, which precludes causal inference; reliance on self-reported measures for behaviors and schedule classification, which may be subject to reporting and social desirability biases; and the absence of precise information on the number of invitations sent and refusals, which precluded calculation of a response rate and may introduce selection bias, thereby limiting the generalizability of the findings. Although the probabilistic sampling strategy reduces the likelihood of systematic selection bias, some degree of nonresponse bias cannot be entirely excluded, as in any email-based survey; if present, such bias would be more likely to affect prevalence estimates than the observed associations. In addition, although the study is nationwide, the absence of explicit geographic stratification may limit the ability to fully capture regional heterogeneity in ICU structure and workforce conditions across Brazil. While multiple covariates were adjusted for, residual and unmeasured confounding may persist (e.g., ICU case mix, staffing ratios, leadership quality). Finally, comparisons with international studies should be interpreted cautiously due to heterogeneity in instruments and burnout thresholds used across the literature.

## CONCLUSION

This nationally representative survey identified a high prevalence of burnout and low life satisfaction among physicians working in Brazilian intensive care units, with significant variation across organizational and labor market characteristics. The analyses indicated consistent associations of workload, fragmented employment arrangements, long commuting distances, and lack of formal intensive care training with poorer well-being indicators. Recovery-related behaviors were associated with more favorable outcomes, but should be interpreted within the structural and organizational context in which these professionals work.

Based on probabilistic sampling and an analytical approach that accounts for multiple employment links per physician, this study provides nationally representative evidence on associations between working conditions and well-being among intensive care unit physicians in Brazil. Given the cross-sectional design, causal inferences cannot be drawn; however, the findings contribute to understanding factors associated with psychological strain in this workforce and support future longitudinal research, national monitoring, and discussions on work organization in intensive care settings.

## References

[1] Moss M, Good VS, Gozal D, Kleinpell R, Sessler CN (2016). An Official Critical Care Societies Collaborative Statement-Burnout syndrome in critical care healthcare professionals: a call for action. Chest.

[2] Papazian L, Hraiech S, Loundou A, Herridge MS, Boyer L (2023). High-level burnout in physicians and nurses working in adult ICUs: a systematic review and meta-analysis. Intensive Care Med.

[3] van Mol MM, Kompanje EJ, Benoit DD, Bakker J, Nijkamp MD (2015). The prevalence of compassion fatigue and burnout among healthcare professionals in intensive care units: a systematic review. PLoS One.

[4] Sanfilippo F, Palumbo GJ, Noto A, Pennisi S, Mineri M, Vasile F (2020). Prevalence of burnout among intensive care physicians: a systematic review. Rev Bras Ter Intensiva.

[5] Chuang CH, Tseng PC, Lin CY, Lin KH, Chen YY (2016). Burnout in the intensive care unit professionals: a systematic review. Medicine (Baltimore).

[6] Azoulay E, De Waele J, Ferrer R, Staudinger T, Borkowska M, Povoa P, ESICM (2020). Symptoms of burnout in intensive care unit specialists facing the COVID-19 outbreak. Ann Intensive Care.

[7] Kerlin MP, McPeake J, Mikkelsen ME (2020). Burnout and joy in the profession of critical care medicine. Crit Care.

[8] Klick JC, Syed M, Leong R, Miranda H, Cotter EK (2023). Health and well-being of intensive care unit physicians: how to ensure the longevity of a critical specialty. Anesthesiol Clin.

[9] Quesada-Puga C, Izquierdo-Espin FJ, Membrive-Jiménez MJ, Aguayo-Estremera R, Cañadas-De La Fuente GA, Romero-Béjar JL (2024). Job satisfaction and burnout syndrome among intensive-care unit nurses: a systematic review and meta-analysis. Intensive Crit Care Nurs.

[10] Shanafelt TD, West CP, Dyrbye LN, Trockel M, Tutty M, Wang H (2022). Changes in burnout and satisfaction with work-life integration in physicians during the first 2 years of the COVID-19 pandemic. Mayo Clin Proc.

[11] Shanafelt TD, West CP, Sinsky CA, Wang H, Carlasare LE, Dyrbye LN (2025). Changes in burnout and satisfaction with work-life integration in physicians and the general US working population between 2011 and 2023. Mayo Clin Proc.

[12] Estenssoro E, Alegría L, Murias G, Friedman G, Castro R, Nin Vaeza N, Latin-American Intensive Care Network (LIVEN) (2017). Organizational issues, structure, and processes of care in 257 ICUs in Latin America: a study from the Latin America Intensive Care Network. Crit Care Med.

[13] Tironi MO, Teles JM, Barros DS, Vieira DF, Silva CM, Martins DF (2016). Prevalência de síndrome de burnout em médicos intensivistas de cinco capitais brasileiras. Rev Bras Ter Intensiva.

[14] Fumis RR, Costa EL, Dal'Col SV, Azevedo LC, Pastore L (2022). Burnout syndrome in intensive care physicians in time of the COVID-19: a cross-sectional study. BMJ Open.

[15] Maslach C, Jackson SE, Leiter MP (2018). Maslach Burnout Inventory Manual.

[16] Diener E, Emmons RA, Larsen RJ, Griffin S (1985). The satisfaction with life scale. J Pers Assess.

[17] Han L, Peng C, Xu Z (2022). The effect of commuting time on quality of life: evidence from China. Int J Environ Res Public Health.

[18] Jung J, Ko K, Park JB, Lee KJ, Cho YH, Jeong I (2023). Association between commuting time and subjective well-being in relation to regional differences in Korea. J Korean Med Sci.

[19] Chen YH, Lin JJ, Yang CW, Tang HM, Jong GP, Yang TY (2024). The effect of commuting time on burnout: the mediation effect of musculoskeletal pain. BMC Health Serv Res.

[20] Trockel MT, Menon NK, Rowe SG, Stewart MT, Smith R, Lu M (2020). Assessment of physician sleep and wellness, burnout, and clinically significant medical errors. JAMA Netw Open.

[21] Kancherla BS, Upender R, Collen JF, Rishi MA, Sullivan SS, Ahmed O (2020). Sleep, fatigue and burnout among physicians: an American Academy of Sleep Medicine position statement. J Clin Sleep Med.

[22] Mincarone P, Bodini A, Tumolo MR, Sabina S, Colella R, Mannini L (2024). Association between physical activity and the risk of burnout in health care workers: systematic review. JMIR Public Health Surveill.

[23] Ruisoto P, Ramírez MR, García PA, Paladines-Costa B, Vaca SL, Clemente-Suárez VJ (2021). Social support mediates the effect of burnout on health in health care professionals. Front Psychol.

[24] West CP, Dyrbye LN, Shanafelt TD (2018). Physician burnout: contributors, consequences and solutions. J Intern Med.

[25] Panagioti M, Panagopoulou E, Bower P, Lewith G, Kontopantelis E, Chew-Graham C (2017). Controlled interventions to reduce burnout in physicians: a systematic review and meta-analysis. JAMA Intern Med.

[26] De Simone S, Vargas M, Servillo G (2021). Organizational strategies to reduce physician burnout: a systematic review and meta-analysis. Aging Clin Exp Res.

[27] Kiratipaisarl W, Surawattanasakul V, Sirikul W (2024). Individual and organizational interventions to reduce burnout in resident physicians: a systematic review and meta-analysis. BMC Med Educ.

[28] Fainstad T, Mann A, Suresh K, Shah P, Dieujuste N, Thurmon K (2022). Effect of a novel online group-coaching program to reduce burnout in female resident physicians: a randomized clinical trial. JAMA Netw Open.

[29] Kiser SB, Sterns JD, Lai PY, Horick NK, Palamara K (2024). Physician coaching by professionally trained peers for burnout and well-being: a randomized clinical trial. JAMA Netw Open.

[30] World Health Organization (WHO) (2019). Burn-out an "occupational phenomenon": International Classification of Diseases.

